# Pregnenolone Inhibits Doxorubicin-Induced Cardiac Oxidative Stress, Inflammation, and Apoptosis—Role of Matrix Metalloproteinase 2 and NADPH Oxidase 1

**DOI:** 10.3390/ph16050665

**Published:** 2023-04-28

**Authors:** Mohamed A. Morsy, Seham A. Abdel-Gaber, Sahar A. Mokhemer, Mahmoud Kandeel, Wael F. Sedik, Anroop B. Nair, Katharigatta N. Venugopala, Hany Ezzat Khalil, Bandar E. Al-Dhubiab, Mervat Z. Mohamed

**Affiliations:** 1 Department of Pharmaceutical Sciences, College of Clinical Pharmacy, King Faisal University, Al-Ahsa 31982, Saudi Arabia; 2 Department of Pharmacology, Faculty of Medicine, Minia University, El-Minia 61511, Egypt; 3 Department of Histology and Cell Biology, Faculty of Medicine, Minia University, El-Minia 61511, Egypt; 4 Department of Biomedical Sciences, College of Veterinary Medicine, King Faisal University, Al-Ahsa 31982, Saudi Arabia; 5 Department of Pharmacology, Faculty of Veterinary Medicine, Kafrelsheikh University, Kafr El-Sheikh 33516, Egypt; 6 Department of Medical Biochemistry, Faculty of Medicine, Minia University, El-Minia 61511, Egypt; 7 Department of Biotechnology and Food Science, Faculty of Applied Sciences, Durban University of Technology, Durban 4000, South Africa; 8 Department of Pharmacognosy, Faculty of Pharmacy, Minia University, El-Minia 61511, Egypt

**Keywords:** pregnenolone, doxorubicin, cardiotoxicity, MMP2, NADPH oxidase 1, caspase-3, inflammation, oxidative stress

## Abstract

The clinical usefulness of doxorubicin (DOX) is limited by its serious adverse effects, such as cardiotoxicity. Pregnenolone demonstrated both anti-inflammatory and antioxidant activity in animal models. The current study aimed to investigate the cardioprotective potential of pregnenolone against DOX-induced cardiotoxicity. After acclimatization, male Wistar rats were randomly grouped into four groups: control (vehicle-treated), pregnenolone (35 mg/kg/d, p.o.), DOX (15 mg/kg, i.p, once), and pregnenolone + DOX. All treatments continued for seven consecutive days except DOX, which was administered once on day 5. The heart and serum samples were harvested one day after the last treatment for further assays. Pregnenolone ameliorated the DOX-induced increase in markers of cardiotoxicity, namely, histopathological changes and elevated serum levels of creatine kinase-MB and lactate dehydrogenase. Moreover, pregnenolone prevented DOX-induced oxidative changes (significantly lowered cardiac malondialdehyde, total nitrite/nitrate, and NADPH oxidase 1, and elevated reduced glutathione), tissue remodeling (significantly decreased matrix metalloproteinase 2), inflammation (significantly decreased tumor necrosis factor-α and interleukin 6), and proapoptotic changes (significantly lowered cleaved caspase-3). In conclusion, these findings show the cardioprotective effects of pregnenolone in DOX-treated rats. The cardioprotection achieved by pregnenolone treatment can be attributed to its antioxidant, anti-inflammatory, and antiapoptotic actions.

## 1. Introduction

Doxorubicin (DOX) is a natural cytotoxic anthracycline antibiotic with a wide range of clinical applications, either as a single agent or in combination with other chemotherapeutics. Common indications include breast, gastric, and hematological malignancies [1,2]. The high incidence of major adverse effects restricts the clinical benefits of DOX, as with most anticancer chemotherapeutics [3]. Importantly, DOX treatment predisposes patients to dose-limiting cardiotoxicity and severe bone marrow suppression. Membrane transporters participate in the accumulation of DOX in cardiomyocytes and, consequently, cellular injury [4]. Given the better economic cost of DOX when compared with other alternatives, finding novel cardioprotective measures is of the utmost benefit to patients with DOX-sensitive malignancies [5,6].

In cancer cells, DOX intercalates with DNA and directly interferes with its transcription and replication. Most importantly, DOX inhibits the activity of topoisomerase II by forming a heterotrimeric complex with DNA and the enzyme, resulting in an accumulation of double-strand DNA breaks and interfering with DNA replication and repair [5,7]. As a result of DNA fragmentation, apoptotic and oxidative stress mechanisms are activated. In addition, DOX and other anthracyclines have been reported to disrupt mitochondrial DNA and metabolic function, further augmenting oxidative stress, inflammatory, and proapoptotic signals [8,9].

Although numerous mechanisms have been postulated and investigated for understanding the cardiotoxic effects of DOX [10,11], current evidence shows that distinct mechanisms are involved in DOX-induced cardiotoxicity other than those underlying its therapeutic effects. The two major factors contributing to DOX-mediated cardiac damage are the alteration of mitochondrial bioenergetics and the increase in cardiomyocytes’ reactive oxygen species (ROS) that are induced by DOX and its metabolites; both processes implicate an interaction with ferrous iron [12,13]. Both DOX and its metabolites, via their quinone moiety, can produce ROS that react with molecular oxygen to create superoxide anion radicals, contributing to DOX-induced DNA damage and the activation of apoptosis. This oxidative-stress-promoting effect of DOX is magnified in the presence of ferrous iron [14], which explains the ability of an exogenous iron chelator to confer protection [5,7]. Endogenous enzymes such as superoxide dismutase and catalase can thus protect cells, at least partially, against the toxic effects of DOX. However, the limited antioxidant capacity of cardiomyocytes compared with other cell types might explain the higher susceptibility to developing cardiotoxicity upon DOX treatment [15]. Moreover, DOX leads to cumulative and dose-dependent cardiotoxicity. For example, at the cumulative dose of 700 mg/m^2^, the percentage of anthracycline-induced cardiotoxicity is 18–48%. Therefore, decreasing the cumulative dose of anthracyclines can lower the incidence of heart failure-associated complications [9].

DOX cardiotoxicity often begins as left ventricular dysfunction and progresses to cardiomyopathy. In addition to cardiotoxicity, DOX has toxic effects on vasculature [16]. It has been proven that DOX increases arterial stiffness by affecting arterial pressure, cardiac function, and perfusion. DOX-induced severe chronic vascular disorder is thought to result from damage to the vascular endothelium via the overproduction of ROS in the mitochondria and the consequent mitochondrial dysfunction [17]. In addition, DOX induces the senescence of vascular smooth muscle cells, thus, playing a role in vascular damage [18]. The rat tail artery, mesenteric artery, and aorta are important models for measuring vascular reactivity [19]. DOX decreases phenylephrine- and endothelin-1-induced contractions and acetylcholine-induced relaxation in aortic segments [20]. Moreover, DOX impairs the contractile responses to the calcium channel activator Bay K 8644 and the protein kinase C activator phorbol 12-myristate 13-acetate in mesangial cells [21]. In addition, with the advancement of monitoring techniques, various cardiac biomarkers, such as creatine kinase isoenzyme (CK-MB), cardiac troponin I, B-type natriuretic peptide, and N-terminal pro-B-type natriuretic peptide (NT-proBNP), have been routinely used in clinical practice to detect DOX-induced cardiomyopathy [22,23].

Pregnenolone is an endogenous steroid that serves multiple functions. In addition to being a key precursor for the synthesis of most steroids, pregnenolone acts as an agonist of the pregnane X receptor (PXR) [24,25] and is an established neurosteroid [26]. In addition to the immunomodulatory effects of pregnenolone, it has certain favorable effects on the hormonal axis. Since pregnenolone is a precursor to steroid hormones, it has been established that reduced pregnenolone serum levels harm the health of male sexuality [27]. Moreover, a reduction in pregnenolone synthesis could directly affect male sperm with respect to hyperactivation [28]. Fortunately, a report indicated that pregnenolone was well-tolerated and did not cause any serious adverse effects. However, pregnenolone treatment for autistic patients has been associated with side effects such as fatigue, diarrhea, and depressive symptoms [29]. Previous reports have illustrated the anti-inflammatory effects of pregnenolone with respect to its interference with the transcriptional activity of inflammatory signals in a PXR-dependent manner [30,31]. Moreover, several reports highlighted the antioxidant effects of pregnenolone [32,33,34]. Based on these anti-inflammatory and antioxidant effects, we hypothesized that pregnenolone would protect the hearts of DOX-challenged rats.

## 2. Results

### 2.1. Pregnenolone Protects against DOX-Induced Cardiac Injury in Rats

**Figure 1:**
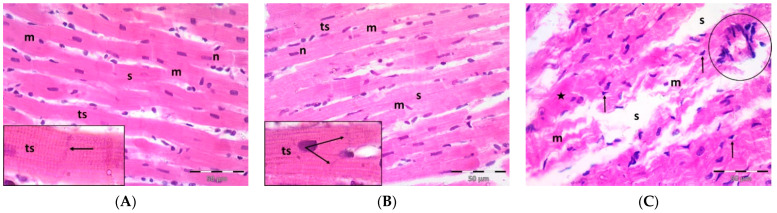

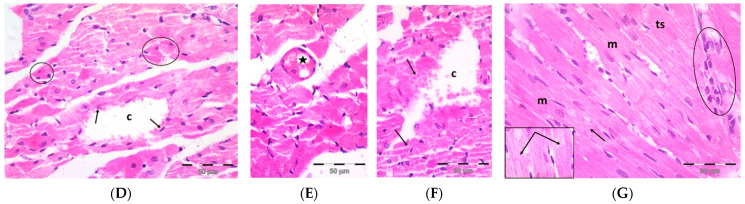
Representative photomicrographs of left ventricular sections (H&E ×400) showing the following: Control (**A**) and pregnenolone-treated control (**B**) groups with longitudinally arranged branched muscle fibers (m) with acidophilic sarcoplasm (s), transverse striations (ts, inset ×1000) and centrally located oval nuclei (n). Notice intercalated disks (arrows, insets ×1000). In contrast, (**C**–**F**) represent the DOX-intoxicated group and show the following: (**C**) degenerated muscle fibers with decompacted corrugated myofibrils (m), pyknotic nuclei (arrows), and inflammatory cell infiltration (circle) and widening of the interstitial spaces (s). Notice loss of transverse striations (star). (**D**) Showing markedly dilated capillaries (c) with endothelial lining (arrows). Notice myocytes with strongly acidophilic sarcoplasm and peripheral pyknotic nuclei (circles). (**E**) Capillary congestion (star) and (**F**) dilated discontinuous capillary (c) with RBC extravasation (arrows). The pregnenolone-treated DOX-intoxicated group (**G**) shows apparently normal muscle fibers (m) with preserved transverse striations (ts) and intercalated disks (arrows, inset ×1000). Notice inflammatory cell infiltration (circle). DOX: doxorubicin.

A single dose DOX challenge (15 mg/kg) in male Wistar rats resulted in obvious cardiac ventricular injury compared with the control animals (Figure 1). The H&E-stained left ventricular tissue sections of the control (Figure 1A) and pregnenolone-treated control (Figure 1B) groups showed a normal longitudinal arrangement of branched muscle fibers (myocytes) with acidophilic sarcoplasm, transverse striations, oval central nuclei, and intercalated disks. On the contrary, the ventricular sections from the DOX-intoxicated group (Figure 1C–F) showed degenerated muscle fibers with decompacted corrugated myofibrils, nuclear pyknosis, inflammatory infiltration, and widening of the interstitial spaces. Additionally, these tissues demonstrated a loss of transverse striations and areas of strongly acidophilic sarcoplasm with peripherally located pyknotic nuclei. Furthermore, unlike the control and pregnenolone-treated control groups, these sections showed markedly dilated and congested capillaries and RBC extravasation. On the other hand, the pregnenolone-treated DOX-intoxicated group (Figure 1G) showed normal muscle fibers, preserved transverse striations, intercalated disks, and some inflammatory cell infiltration. The results of the histopathological scoring are presented in Table 1.

**Figure 2:**
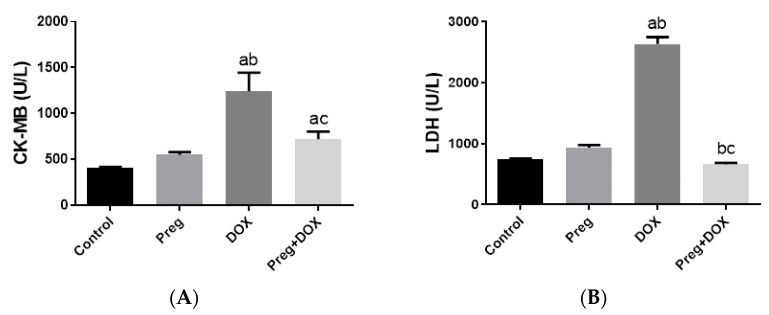
Serum levels of CK-MB (**A**) and LDH (**B**) in DOX-induced cardiotoxicity in rats. Results show the mean of six observations ± SEM. ^a,b,c^ Significant difference (*p* < 0.05) compared to control, Preg control, and DOX groups, respectively. CK-MB: creatine kinase MB; LDH: lactate dehydrogenase; Preg: pregnenolone; DOX: doxorubicin.

Parallel to the histopathological findings, the untreated DOX-intoxicated rats showed the highest serum values of CK-MB (1243 ± 81.76 U/L) and lactate dehydrogenase (LDH) (2633 ± 114.4 U/L) when compared with the DOX-untreated vehicle-treated or pregnenolone-treated rats, which showed the lowest values (*p* < 0.05) (Figure 2). In rats that received pregnenolone for seven consecutive days and were challenged with DOX on the fifth day (group 4), the serum levels of CK-MB (719.5 ± 33.60 U/L) and LDH (661.7 ± 25.40 U/L) were significantly lower than those observed in the untreated DOX group (*p* < 0.05).

### 2.2. Pregnenolone Prevents DOX-Induced Myocardial Oxidative Stress

**Figure 3:**
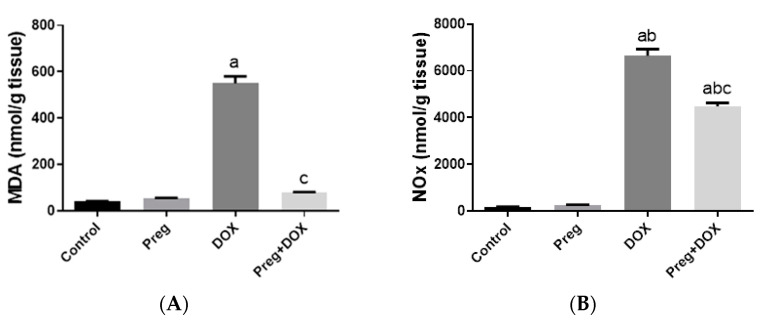

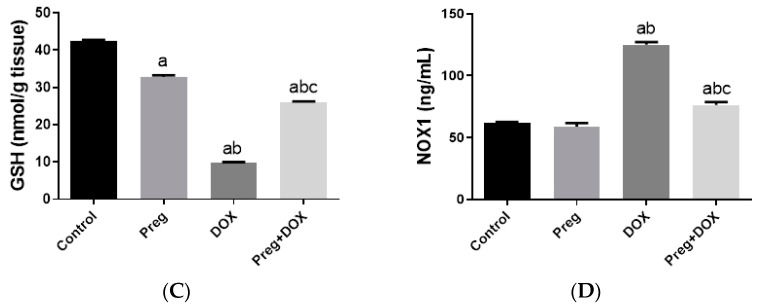
Myocardial levels of MDA (**A**), NOx (**B**), GSH (**C**), and NOX1 (**D**) after DOX and/or Preg administration. Results show the mean of six observations ± SEM. ^a,b,c^ Significant difference (*p* < 0.05) compared to control, Preg control, and DOX groups, respectively. MDA: malondialdehyde; NOx: total nitrite/nitrate; GSH: reduced glutathione; NOX1: NADPH oxidase 1; Preg: pregnenolone; DOX: doxorubicin.

To investigate the effect of the DOX-induced upregulation of oxidative stress and its role in cardiac damage, we measured the myocardial content of malondialdehyde (MDA), a lipid peroxidation end-product; the total nitrite/nitrate (NOx) content, as an indicator of nitric oxide (^•^NO) metabolism; and reduced glutathione (GSH), as a marker of endogenous cardiac antioxidant capacity. Compared with the control groups (groups 1 and 2), the untreated DOX-challenged hearts in group 3 showed dramatic increases (*p* < 0.05) in MDA and NOx levels, which were accompanied by a substantial reduction (*p* < 0.05) in GSH levels (Figure 3). On the other hand, the cardiac tissues from the pregnenolone-treated DOX-intoxicated rats in group 4 showed normalized levels of MDA and were showed GSH levels closer to normal when compared with the DOX-treated group. Furthermore, when compared to rats in group 3 (untreated DOX rats, Figure 3C), their NOx concentrations significantly decreased after pregnenolone therapy. On the other hand, levels of NADPH oxidase 1 (NOX1), a ROS-generating enzyme, in cardiac tissue homogenates were measured. The pregnenolone-treated DOX-intoxicated rats in group 4 displayed almost normal NOX1 levels after their increase via DOX administration (Figure 3D).

### 2.3. Pregnenolone Protects against DOX-Induced Cardiac Remodeling and Inflammation

**Figure 4:**
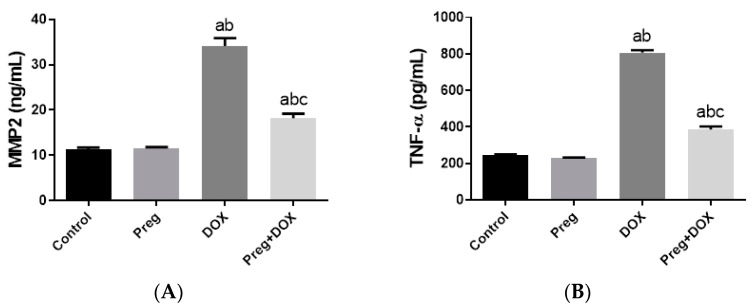
Expression of MMP2 (**A**) and TNF-α (**B**) in cardiomyocytes of rats after DOX and/or Preg administration. Results show the mean of six observations ± SEM. ^a,b,c^ Significant difference (*p* < 0.05) compared to control, Preg control, and DOX groups, respectively. MMP2: matrix metalloproteinase 2; TNF-α: tumor necrosis factor-alpha; Preg: pregnenolone; DOX: doxorubicin.

**Figure 5:**
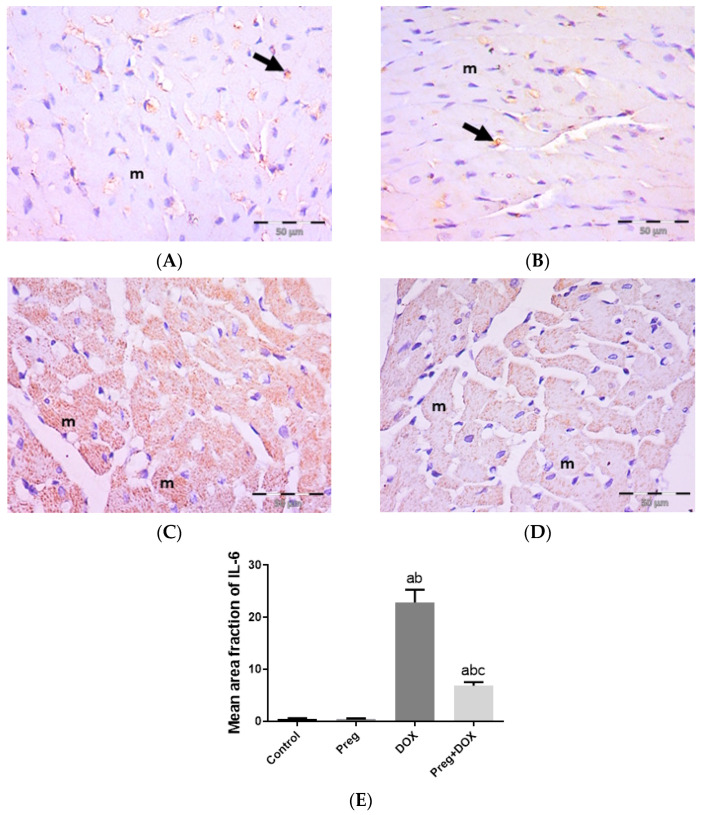
Representative immunohistochemical photomicrographs (×400) of left ventricular sections stained for IL-6, showing the following: control (**A**) and Preg-treated control (**B**) groups with negative myocyte expression (m). Notice non-specific reactions in intercellular spaces (arrows). The DOX-intoxicated group (**C**) shows strong positive IL-6 cytoplasmic expression in most myocytes (m). The Preg-treated DOX-intoxicated group (**D**) shows myocytes with faint IL-6 cytoplasmic expression (m). (**E**) Surface area fraction of IL-6 reactivity. Results are expressed as the mean ± SEM of 6 fields in each group. ^a,b,c^ Significant difference (*p* < 0.05) compared to control, Preg control, and DOX groups, respectively. IL6: interleukin 6; Preg: pregnenolone; DOX: doxorubicin.

Figure 4A demonstrated that following DOX treatment, matrix metalloproteinase 2 (MMP2) levels in the heart were higher than they were in the control groups. Rats treated with pregnenolone had significantly lower levels than the DOX-treated rats. Further, we evaluated myocardial inflammation after DOX administration in all experimental groups by measuring tumor necrosis factor-alpha (TNF-α) levels in cardiac tissue homogenates (Figure 4B) and interleukin 6 (IL-6) expression in ventricular tissue sections (Figure 5). Both the vehicle- and pregnenolone-treated control rats showed basal cardiac levels of TNF-α. On the contrary, the untreated DOX-intoxicated rats showed the highest cardiac TNF-α levels, indicating increased tissue inflammation. Importantly, pretreating the rats with pregnenolone significantly attenuated the DOX-induced cardiac inflammation (Figure 4B). However, pregnenolone did not completely inhibit the DOX-induced induction of TNF-α; these rats still showed significantly higher levels of the inflammatory cytokine than the control groups (groups 1 and 2). In addition, IL-6 immunohistochemical expression in left ventricular tissue sections revealed negative myocyte expression in both the control (Figure 5A) and pregnenolone-treated control (Figure 5B) groups. On the other hand, the DOX-intoxicated group (Figure 5C) showed diffuse, strong, positive cytoplasmic expression with a significant increase in the area fraction of IL-6 immunoreactivity compared to both the control and pregnenolone-treated control groups; however, the pregnenolone-treated DOX-intoxicated group (Figure 5D) showed faint expression with a significant decrease in the area fraction of IL-6 compared to the DOX-intoxicated group but still significantly higher than both control groups (Figure 5E).

### 2.4. Pregnenolone Protects against DOX-Induced Activation of Apoptosis

**Figure 6:**
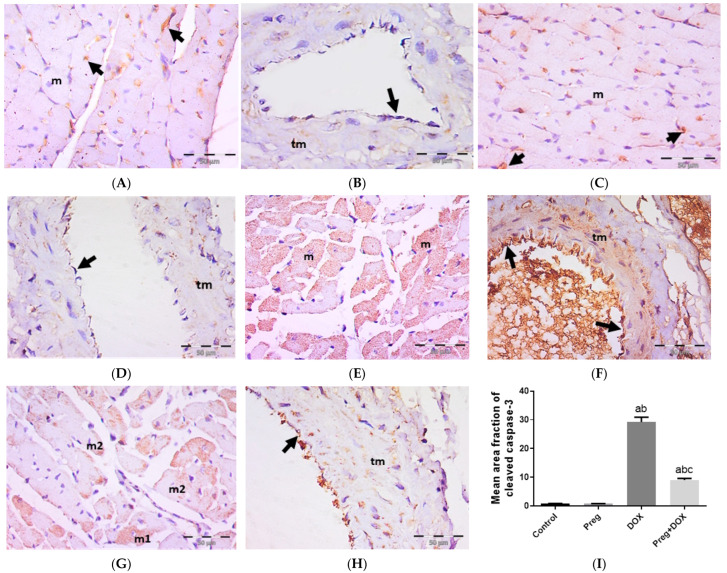
Representative immunohistochemical photomicrographs (×400) of left ventricular sections stained for cleaved caspase-3, showing control (**A**,**B**) and Preg-treated control (**C**,**D**) groups. (**A**,**C**) Negative expression in myocytes (m). Notice non-specific reactions in intercellular spaces (arrows). (**B**,**D**) Representing coronary vessels with negative expression in vascular endothelium (arrows) and tunica media (tm). The DOX-intoxicated group (**E**,**F**) shows positive cytoplasmic expression in most of the myocytes (m) (**E**), and vascular endothelium (arrows) and tunica media (tm) of coronary vessels (**F**). The pregnenolone-treated DOX-intoxicated group (**G**,**H**) showing (**G**) scattered myocytes with positive cleaved caspase-3 cytoplasmic expression occupying either the whole sarcoplasm (m1) or the subsarcolemmal sarcoplasm (m2). (**H**) Representing coronary vessels with positive expression in vascular endothelium (arrow). Notice minimal expression in tunica media (tm). (**I**) Surface area fraction of cleaved caspase-3 reactivity. Results are expressed as the mean ± SEM of 6 fields in each group. ^a,b,c^ Significant difference (*p* < 0.05) compared to control, Preg control, and DOX groups, respectively. Preg: pregnenolone; DOX: doxorubicin.

We tested the ability of pregnenolone treatment to inhibit DOX-activated apoptotic changes in myocardial tissues by measuring the tissue expression of cleaved caspase-3 (Figure 6). The results showed a negative expression of cleaved caspase-3 in both cardiac myocytes (Figure 6A,C) and coronary vessels (Figure 6B,D) in both the control and pregnenolone-treated control groups. On the contrary, sections from the DOX-intoxicated group showed positive cytoplasmic expression in most of the cardiac myocytes (Figure 6E) and vascular endothelium and the tunica media of the coronary vessels (Figure 6F) after being challenged with a single dose of DOX (15 mg/kg), with a significant increase in the cleaved caspase-3 mean area fraction compared to both control groups. This activation of apoptosis by DOX was inhibited by pretreating the rats with pregnenolone in the pregnenolone-treated DOX-intoxicated group. Some myocytes revealed negative expression, and others showed subsarcolemmal cytoplasmic expression; however, few myocytes showed positive expression occupying the whole sarcoplasm (Figure 6G). Coronary vessels revealed positive vascular endothelium expression with minimal tunica media expression (Figure 6H). The cardioprotective effect of pregnenolone was proved morphometrically via the significant decrease in the cleaved caspase-3 mean area fraction in the pregnenolone-treated DOX-intoxicated group compared to the DOX-intoxicated group but with a significant difference compared to both control groups (Figure 6I).

## 3. Discussion

The most severe side effect of DOX therapy is dose-dependent cardiotoxicity, which finally results in cardiomyopathy and significantly restricts the clinical use of DOX. Therefore, there is a growing need for cardiac scores to predict antineoplastic-induced cardiomyopathies to provide early diagnosis and therapy initiation in the same way as the electrocardiographic diastolic index, a simple formula that seems to have a prominent role in indicating diastolic dysfunction [35]. On the other hand, there is a real need for novel therapeutic approaches to treat DOX-induced cardiotoxicity. Pregnenolone is a neurosteroid that has been shown to have beneficial effects on other tissues in addition to its ability to reduce inflammation in the brain [31,34,36]. In this study, we sought to identify a further potential function of pregnenolone in reducing the cardiac toxicity of the chemotherapeutic drug DOX. Our findings demonstrated that pregnenolone treatment was effective in reducing the main paths proposed for DOX-induced cardiomyocyte damage, thus highlighting the possible protective benefits of pregnenolone in combination with DOX.

The model of DOX-induced cardiotoxicity was established at a single i.p. dose of 15 mg/kg in Wistar rats [37,38]. Despite this, the rat i.p. LD_50_ of DOX is 16 mg/kg [39]. However, it is well-known that parameter variations between experiments, such as animal weight and strain, the time of administration, and animal care maintenance, critically affect the LD_50_. According to the data presented herein, DOX-induced cardiomyocyte damage was evident in the disrupted left ventricular structure, discontinuous capillaries denoting endothelial damage, and higher serum levels of CK-MB and LDH, in line with others [40,41]. Namely, the CK-MB isoenzyme is a reliable marker for cardiac damage [42]. It controls the synthesis and uptake of high-energy phosphates within cardiac tissues. Moreover, it plays a more general role in transporting high-energy phosphate bonds via creatine phosphate from the mitochondrial site of ATP production to the cytoplasmic site of use [22]. When the cellular wall of a cardiomyocyte is damaged, the serum levels of the intracellular enzyme CK-MB increase. Conversely, pregnenolone restored the cardiomyocyte’s natural architecture while protecting the cellular structure and minimizing cardiac enzyme leakage. Here, we show that a variety of variables associated with DOX-induced cardiotoxicity are responsible for pregnenolone’s cardioprotective effects.

The current findings showed that the damage caused by DOX was mostly due to the production of ROS, which destroy cell walls and trigger apoptosis. Many studies confirmed our findings that DOX-induced heart damage is primarily caused by increased oxidative/nitrosative stress linked with a reduced antioxidant defense state [12,40,43,44,45,46]. Increased cardiac lipid peroxidation and decreased cardiac cellular GSH content in DOX-treated rats are indicators of an imbalanced redox state in the cardiomyocyte. Pregnenolone, on the other hand, replenishes the cardiomyocyte GSH and lessens the oxidative damage that is visible in the heart tissue by lowering lipid peroxidation. This antioxidant property has been reported to guard against DOX-induced cardiotoxicity [47].

It is still debatable how ^•^NO contributes to the cardiotoxicity caused by DOX. Increased ^•^NO has been shown to both improve and defend against cardiac toxicity induced by DOX [43,48]. Our findings appear to be in agreement with the increase in ^•^NO caused by DOX, which denotes nitrative stress. The nitrative damage in the heart tissue was lessened in the pregnenolone-treated rats. As ^•^NO combines with the superoxide anion, peroxynitrites are produced, which are known to damage DNA and cause an energy imbalance and eventual cell death [40,43].

NOXs are a class of plasma-membrane-connected enzymes that serve to generate ROS. The ROS produced by NOX are crucial signaling pathway modulators that regulate important physiological processes. However, the excessive and persistent release of ROS produced from NOX has a deleterious impact on cells [49]. Following DOX therapy, NOX contributes to the production of ROS, whereas DOX can stimulate NOX activation. Moreover, it has been proven that NOX-derived ROS are responsible for the cardiac apoptosis caused by DOX [50,51,52]. Here, we observed that rats treated with DOX had greater NOX1 levels, which is consistent with earlier studies [50,51,52]. On the other hand, pregnenolone was able to decrease NOX1 levels while also reducing oxidative damage. Moreover, increased heart dysfunction, remodeling, and fibrosis are caused by the upregulation of NOX1 in DOX cardiotoxicity through the stimulation of cardiac fibroblasts’ pro-fibrotic response, such as MMP activation [52].

It is widely known that the extracellular matrix proteins are proteolyzed by MMPs prior to the deposition of microfilaments in tissue lesions, including myocardial infarction [53]. The degradation of contractile apparatus elements by MMP2, such as light chain myosin 1, results in a decrease in heart contractility. Consequently, MMP2 has been regarded as a biomarker of cardiac remodeling in patients with large regions of cardiac damage [54]. It has been shown that early in cardiotoxicity, the oxidative stress brought on by DOX activates MMP2, which is the most frequent MMP in cardiomyocytes [55]. In accordance with the results indicated above, herein, pregnenolone was able to decrease DOX-induced ROS production and its subsequent effect on MMP2 expression in the cardiac tissue. Therefore, the ability of pregnenolone to lessen the level of MMP2 is considered a therapeutic modality for DOX-induced cardiotoxicity in line with others [51,55].

Proinflammatory cytokines such as TNF-α and IL-6, acting directly on the myocardium at a later stage of DOX-induced cardiac injury, generate additional oxidative/nitrosative stress with hypertrophy and fibrosis, ultimately leading to heart failure [56,57]. In the current investigation, the increase in both markers in the cardiomyocytes of the DOX-treated rats suggested that the inflammatory response had been activated, in accordance with previous evidence [56,57]. When administered concurrently with DOX, pregnenolone’s inhibition of the cytokines TNF-α and IL-6 indicates its anti-inflammatory activity, which has been thoroughly researched in bone and other tissues as well as the brain [31,34,36]. Furthermore, TNF-α drives the extrinsic apoptotic pathway induced by DOX in cardiomyocytes. When TNF-α functions as a death ligand by binding to its receptors, cytosolic proteins are recruited to activate caspase 8, and activated caspase 8 can activate caspase-3 to cause apoptosis [58].

One of the mechanisms of DOX-induced cardiotoxicity is the activation of caspase-3, which causes the apoptosis of cardiomyocytes [40]. In the current work, the presence of the activated caspase-3 form, cleaved caspase-3, in the cytoplasm of the cardiomyocytes of rats receiving DOX is a sign that these myocytes have undergone apoptosis. The expression of cleaved caspase-3 has, on the other hand, been inhibited by pregnenolone. This antiapoptotic potential for pregnenolone was described previously in primary cortical neurons. Pregnenolone inhibits cortical neuronal apoptosis and degeneration brought on by staurosporine and glutamate by increasing ERK-MAPK phosphorylation when present in physiological quantities [59]. In addition, pregnenolone was shown to be able to cause apoptosis in glioma cells via a caspase-3-dependent mechanism, which is contrary to earlier findings [60]. However, the ability of pregnenolone to suppress or promote apoptosis may rely on the concentration of pregnenolone in cells and the type of cells, whether normal or malignant.

The current findings may have significant clinical implications for reducing the inflammation and cardiotoxicity caused by DOX. The excellent tolerability profile of pregnenolone is a motivating element for future research to highlight its positive effects and identify any other negative side effects when it is administered prophylactically with DOX therapy. In addition, the dose selected in the current study was relevant to the human dose used safely in neurological diseases [61].

## 4. Materials and Methods

### 4.1. Experimental Animals and Study Design

Male Wistar rats weighing 180–200 g were procured from the National Research Center (NRC, Giza, Egypt) and housed under standard conditions. The rats had free access to regular animal food and water. The Faculty of Medicine Research Ethics Committee, Minia University, Egypt, approved all animal experiments (ethical approval No. 531/2022). The Institutional Review Board of the Faculty of Medicine, Minia University is constituted and operating according to the Declaration of Helsinki, CIOMS, ICH-GCP guidelines, and Egyptian law No. 214/2020.

**Figure 7:**
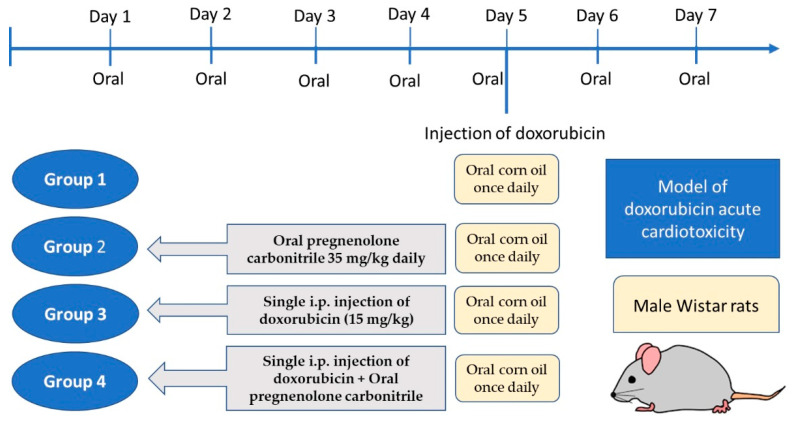
Timeline schedule of the treatment regimen.

After acclimatization for one week, twenty-four rats were randomly divided into four groups (six rats each). Group 1 received oral corn oil once daily for seven days; group 2 received pregnenolone carbonitrile (Cas Number: 1434-54-4, Toronto Research Chemicals, Toronto, ON, Canada) as a single oral daily oral dose of 35 mg/kg dissolved in corn oil for seven days; group 3 received a single i.p. injection of DOX (15 mg/kg) on the fifth day of experiment, in addition to daily oral corn oil as in group 1; and group 4 received the combined treatment of pregnenolone and DOX, as described for groups 2 and 3, respectively (Figure 7). The DOX dose used in the current study was based on our pilot study and previous publications [37,38]. A short-term rat model receiving a high-dose DOX injection would be appropriate for assessing acute cardiotoxicity [62]. However, a limitation of the current study is that for the verification of myocardial damage, we used biochemical and histological markers but not echocardiography. The dose of pregnenolone carbonitrile was chosen according to a previous publication [63]. The dose of pregnenolone is safe as its LD_50_ is 100 mg/kg in rats, according to the safety data sheet. Moreover, its safety was approved via dose translation between laboratory rats and humans to predict the human equivalent dose [64].

### 4.2. Sample Collection

One day after the final treatment, the rats were anesthetized with thiopental sodium (50 mg/kg, i.p.), and their blood was collected by cardiac puncture and used for serum isolation. The hearts were immediately harvested and washed in cold saline, and a portion was flash-frozen in liquid nitrogen and stored for biochemical assay. The other portion (the left ventricles) was fixed in 10% neutral buffered formalin and used for histopathology and immunohistochemistry [49,50].

### 4.3. Determination of Serum CK-MB, LDH, and Redox State

The serum activity of the cardiac function markers CK-MB and LDH was determined using an ELISA assay (CAT#E0311Ra, Bioassay Technology Laboratory, Birmingham, UK) and a commercial LDH kit (Biomed Diagnostics, Badr City, Egypt), respectively, and measured as previously reported, according to the manufacturer’s instructions [65].

The concentrations of MDA, GSH, and NOx were determined in ventricular tissue homogenates (10% *w*/*v* in phosphate-buffered saline) via spectrophotometry using a Beckman DU-64 UV/VIS spectrophotometer. As a lipid peroxidation marker, MDA reacts with thiobarbituric acid in an acidic medium to form a colored adduct with maximal absorbance at 534 nm; the measured values were proportional to the amount of lipid peroxides [66]. Cardiac GSH was determined based on its ability to react with 5,5′-dithio-bis-2-nitrobenzoic acid (Ellman’s reagent) and form a yellow product [65,67]. Tissue NOx was determined as an indicator of cardiac ^•^NO content. A copper-coated cadmium reagent was used to reduce nitrate into nitrite. In an acid medium, nitrite reacts with the Griess reagent to form a pink-colored product; the concentration was measured spectrophotometrically, as previously reported [66].

### 4.4. Determination of Cardiac NOX1, MMP2, and TNF-α Levels

The levels of cardiac NOX1, MMP2, and TNF-α were estimated in the supernatants of the rat ventricular tissue homogenates using rat ELISA kits for TNF-α (RAB0480, Sigma-Aldrich, St. Louis, MO, USA), MMP2 (GWB-ZZD134, Genway Biotech, San Diego, CA, USA), and NOX1 (MBS2702015, MyBiosource, Inc., San Diego, CA, USA) according to the manufacturers’ instructions [68].

### 4.5. Histopathology and Immunohistochemistry

Formalin-fixed, paraffin-embedded heart tissue sections (5-µm thick) from the left ventricle were stained with H&E according to the standard procedure to study the structure of the cardiac muscle in each group [69].

For the immunohistochemical assessment of cardiac IL-6 and cleaved caspase-3, the sections were rehydrated, washed with phosphate-buffered saline (PBS), and treated with 3% H_2_O_2_ for blocking endogenous peroxidases. Sections were placed into EDTA buffer for antigen retrieval, and nonspecific binding was then blocked by 5% bovine serum albumen. Sections were then incubated overnight at 4 °C with the primary antibodies: polyclonal anti-cleaved caspase-3 (catalog number: Asp175, Cell Signaling Technology, Danvers, MA, USA) at a concentration of 1/200 and polyclonal anti-IL-6 (catalog number: GTX54672, GeneTex, Irvine, CA, USA) at a concentration of 1/100. Next, the sections were washed with PBS and incubated with an anti-rabbit secondary antibody with conjugated peroxidase. The expression of the target proteins was monitored by the brown color formed when 3,3′-diaminobenzidine (DAB) was oxidized by peroxidase in the presence of hydrogen peroxide. The cardiac sections were then counterstained with hematoxylin, dehydrated by alcohol, cleared by xylene, and mounted [70]. A negative control (the same immunohistochemical staining protocol without incubation with the primary antibodies) was performed to eliminate nonspecific binding with the secondary antibody.

The presence and severity of histological abnormalities (distorted architecture, loss of muscle striation, presence of pyknotic nuclei, vascular congestion, hemorrhage, and inflammatory cell infiltration) were assessed as follows: (−) for the absence of abnormalities, (+) for mild abnormalities, (++) for moderate abnormalities, or (+++) for severe abnormalities [71,72]. The mean area fraction of anti-IL-6 and anti-cleaved caspase-3 immunoreactivity were assessed using the software image J, version 1.53c (http://rsbweb.nih.gov/ij/; NIH, Bethesda, MD, USA, accessed on 1 January 2023). Images were acquired using an Olympus digital camera (LC20, Olympus Co., Tokyo, Japan) coupled to an Olympus light microscope (BX51, Olympus Co.). Six non-overlapping fields at the magnification of 400 from each rat of each group were randomly selected and assessed for histological scoring and image analysis [73]. The histologist was unaware of the study group assignments.

### 4.6. Data Analysis

The results (mean ± SEM) were analyzed with the help of GraphPad Prism, version 8.0.0, for Windows (GraphPad Software, San Diego, CA, USA). Comparisons between different experimental groups’ mean values were carried out using Tukey’s post hoc test following s one-way analysis of variance (ANOVA). At *p* value < 0.05, the differences between means were considered significant. The Kolmogorov–Smirnov test was used to determine the normality for each group across all parameters, and the results were insignificant, with a *p* value > 0.05.

## 5. Conclusions

The current investigation showed for the first time that pregnenolone has an antioxidant effect that is efficient at the reducing oxidative and nitrative damage caused by DOX in the cardiomyocyte. Moreover, pregnenolone has actions that counteract the cardiotoxicity caused by DOX by reducing inflammation, remodeling, and apoptosis.

## Figures and Tables

**Table 1:** The histopathological scoring of the cardiac injury.

Histological Changes	Control	Preg	DOX	Preg + DOX
Distorted architecture of cardiac muscles	−	−	+++	+
Loss of muscular striations	−	−	++	+
Cells with pyknotic nuclei	−	−	+++	+
Vascular congestion	−	−	+++	+
Hemorrhage	−	−	++	-
Inflammatory cellular infiltrate	−	−	++	+

Score (−) is considered normal. Scores (+), (++), and (+++) are mild, moderate, and severe abnormalities, respectively. Preg: pregnenolone; DOX: doxorubicin.

## Data Availability

Data is contained within the article.
